# Mitochondrial-nuclear epistasis underlying phenotypic variation in breast cancer pathology

**DOI:** 10.1038/s41598-022-05148-4

**Published:** 2022-01-26

**Authors:** Pierre R. Bushel, James Ward, Adam Burkholder, Jianying Li, Benedict Anchang

**Affiliations:** 1 Massive Genome Informatics Group, National Institute of Environmental Health Sciences, 111 T.W. Alexander Drive, P.O. Box 12233, Research Triangle Park, NC 27709 USA; 2 Biostatistics and Computational Biology Branch, National Institute of Environmental Health Sciences, Research Triangle Park, NC 27709 USA; 3 Integrative Bioinformatics Group, National Institute of Environmental Health Sciences, Research Triangle Park, NC 27709 USA; 4 Kelly Government Solutions, Research Triangle Park, NC 27709 USA; 5 Office of Environmental Science Cyberinfrastructure, National Institute of Environmental Health Sciences, Research Triangle Park, NC 27709 USA

**Keywords:** Epistasis, Genetic association study

## Abstract

The interplay between genes harboring single nucleotide polymorphisms (SNPs) is vital to better understand underlying contributions to the etiology of breast cancer. Much attention has been paid to epistasis between nuclear genes or mutations in the mitochondrial genome. However, there is limited understanding about the epistatic effects of genetic variants in the nuclear and mitochondrial genomes jointly on breast cancer. We tested the interaction of germline SNPs in the mitochondrial (mtSNPs) and nuclear (nuSNPs) genomes of female breast cancer patients in The Cancer Genome Atlas (TCGA) for association with morphological features extracted from hematoxylin and eosin (H&E)-stained pathology images. We identified 115 significant (q-value < 0.05) mito-nuclear interactions that increased nuclei size by as much as 12%. One interaction between nuSNP rs17320521 in an intron of the WSC Domain Containing 2 (*WSCD2*) gene and mtSNP rs869096886, a synonymous variant mapped to the mitochondrially-encoded NADH dehydrogenase 4 (*MT-ND4*) gene, was confirmed in an independent breast cancer data set from the Molecular Taxonomy of Breast Cancer International Consortium (METABRIC). None of the 10 mito-nuclear interactions identified from non-diseased female breast tissues from the Genotype-Expression (GTEx) project resulted in an increase in nuclei size. Comparisons of gene expression data from the TCGA breast cancer patients with the genotype homozygous for the minor alleles of the SNPs in *WSCD2* and *MT-ND4* versus the other genotypes revealed core transcriptional regulator interactions and an association with insulin. Finally, a Cox proportional hazards ratio = 1.7 (C.I. 0.98–2.9, p-value = 0.042) and Kaplan–Meier plot suggest that the TCGA female breast cancer patients with low gene expression of *WSCD2* coupled with large nuclei have an increased risk of mortality. The intergenomic dependency between the two variants may constitute an inherent susceptibility of a more severe form of breast cancer and points to genetic targets for further investigation of additional determinants of the disease.

## Introduction

Breast cancer has an enormous burden on the afflicted and other than heart disease, it is among the leading causes of death in women. Investigations have uncovered germline mutations and somatic variations that contribute to the etiology of breast cancer^1^. Recently, there has been attention paid to the contribution of mitochondrial genome variants in the predisposition of breast cancer^2^. However, little is known about the interplay of mitochondrial single nucleotide polymorphisms (mtSNPs) with nuclear SNPs (nuSNPs) in modifying phenotypes associated with breast cancer.

In this investigation, mitochondrial-nuclear (mito-nuclear) SNPs were associated with morphological features extracted from images of hematoxylin and eosin (H&E) stained slides of breast cancer tissue from 286 female subjects in The Cancer Genome Atlas (TCGA)^3^ and 259 non-diseased breast tissues from the Genotype-Expression (GTEx) project^4^. Significant mito-nuclear interactions only in the TCGA cohort were confirmed using genotype and morphological features extracted from 523 Molecular Taxonomy of Breast Cancer International Consortium (METABRIC) cohort^5^ breast cancer tissue samples. Of the 115 statistically significant mito-nuclear interactions found in the TCGA data set, one nuSNP in an intron of WSC Domain Containing 2 (*WSCD2*) was found to interact with a mtSNP in the mitochondrially-encoded NADH dehydrogenase 4 (*MT-ND4*) gene leading to a significant increase of as much as ~ 12% in the mean size of nuclei and was confirmed in the METABRIC breast cancer tissue cohort as an independent data set. None of the significant interactions were found in the non-diseased cohort. The hope is the mito-nuclear interaction that associates with the increase in breast cancer nuclei size will be of interest to investigators to determine dysregulated biological pathways manifested from coordinated mito-nuclear epistasis which would potentially serve as molecular targets for new therapeutic interventions and opportunities for devising a cure of the disease.

## Results

### Mito-nuclear interactions affect nuclei size

**Figure 1:**
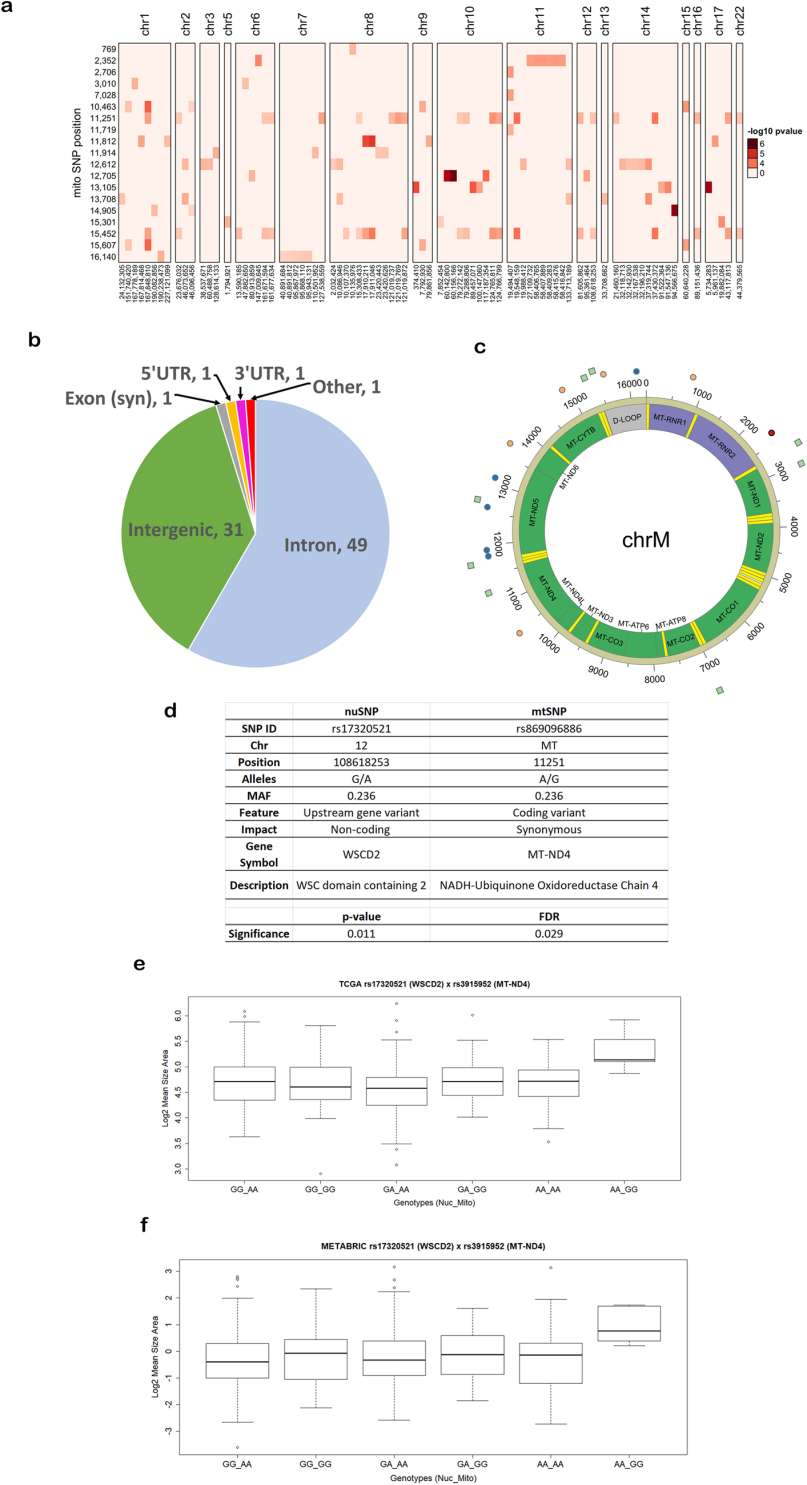
Significant mito-nuclear interactions. (**a**) Shown is a heat map of -log10 p-values ordered by mitochondrial genome position on the y-axis and nuclear genome position (chromosome, position) on the x-axis. All p-values shown meet the FDR q-value threshold for statistical significance of < 0.05 and Bonferroni p-value < 0.05. The Heatmap was produced in R using ComplexHeatmap. (**b**) Genomic targets of nuSNPs. Syn: synonymous substitution, UTR: untranslated region. (**c**) Circos plot of the mitochondrial genome (chrM) with genes color coded and labeled and tRNAs denoted as yellow bands. Points outside of the genome represent the variant allele frequency (VAF) of the mtSNPs. Red circle: VAF < 0.1, Blue circle: 0.1 ≥ VAF < 0.2, Orange circle: 0.2 ≥ VAF < 0.3, Green square: VAF ≥ 0.3. (**d**) Genomic information for the interacting SNPs significant in TCGA and METABRIC cohorts. The p-value and false discovery rate (FDR) q-value are from the TCGA data analysis. (**e**) Boxplot of TCGA log2 mean size area normalized by number of nuclei (y-axis) for the nuSNP rs17320521 (WSCD2) by mtSNP rs869096886 (MT-ND4) interaction. The x-axis is the genotypes for the nuSNP by mtSNP allele pairs. (**f**) Same as (**e**) except for METABRIC data.

Physicians and pathologists evaluate the size, shape and intensity of nuclei in biopsies of breast tissues as some of the characteristics for grading the severity of breast cancer. In this investigation, nuclear morphological features (nuclei number, nuclei size area, nuclei intensity and nuclei circularity) were extracted from images of hematoxylin and eosin (H&E) stained slides of 286 female patients breast cancer tissue in TCGA^3^ (Supplementary Data 1), 523 female breast cancer tissue samples (Supplementary Data 2) from METABRIC^5^ and non-diseased breast tissues from 259 donors in the GTEx project (Supplementary Data 3)^4^. The majority of the TCGA patients (n = 222) had a primary diagnosis of infiltrating ductal carcinoma. We focused on ~ 12,400 nuclear SNPs in or near genes that encode mitochondrial proteins to assess the joint effect with mtSNPs on the morphological features. In a mixed linear model, resemblance of the individuals was accounted for by incorporating a kinship matrix as a random effect and population structure was adjusted by including the first five principal components (PCs) from the principal component analysis of the genotype data. From the scree plot (Supplementary Fig. 1), the elbow of the graph suggests that the first five PCs are sufficient to capture the cumulative variance of the population structure for the TCGA patients. A similar scree plot was generated for the GTEx data indicating that the first five PC are sufficient to account for population structure (Supplementary Fig. 2). The METABRIC cohort did not have demographics on the race of the individuals. At q-value < 0.05 as a threshold of significance and post hoc test of the multiple pairwise comparisons of the means of the phenotypes for each genotype pair (FDR < 0.05), we identified 115 mito-nuclear interactions (Supplementary Data 4) genetically linked to increase in nuclei size within the TCGA patients (Fig. 1a). The majority of the nuSNPs from the significant interactions map to introns of genes or intergenic regions (Fig. 1b). However, one nuSNP (synonymous substitution) maps to an exon of a gene and two other ones in untranslated region of genes. Figure 1c illustrates the location and variant allele frequencies of the mtSNPs from the significant interactions. Ten mito-nuclear interactions (Supplementary Data 5) were significantly associated with the nuclei size morphological feature extracted from the female donors in GTEx. However, given the GTEx donors with minor alleles for the SNPs in the two genomes, none of the 10 mito-nuclear interactions increased the nuclei size in comparison to the other genotypes.

### Confirmation using an independent data set

To confirm the significant associations of the mito-nuclear interactions with increased nuclei size in the TCGA data set, we analyzed the genotype data and morphological features extracted from images of H&E stained slides of breast cancer tissue from the female subjects in the METABRIC cohort as an independent data set. The majority of the subjects (n = 422) had a primary diagnosis of invasive ductal carcinoma. As shown in Fig. 1d, a significant mito-nuclear SNP interaction overlapped with the TCGA hits (TCGA q-value = 0.029, METABRIC q-value 0.043). The nuSNP rs17320521 in an intron of the WSC Domain Containing 2 (*WSCD2*) gene interaction with mtSNP rs869096886 (a synonymous variant mapped to the mitochondrially-encoded NADH dehydrogenase 4 (*MT-ND4*) gene) is significantly associated with as much as a 12% increase in the size of the nuclei (Fig. 1e,f).

**Figure 2:**
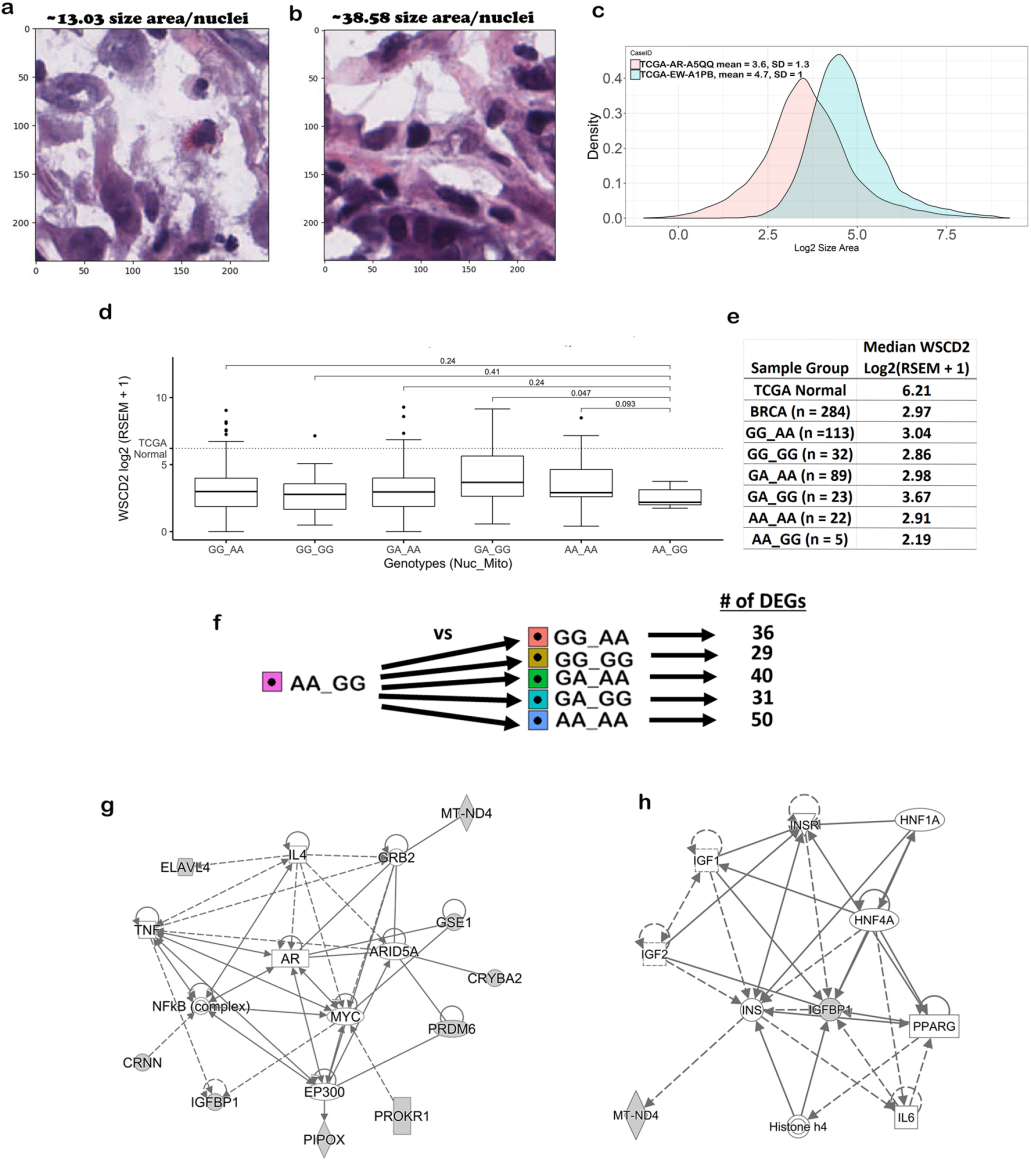
Biological impact of the significant mito-nuclear interaction. (**a**) Hematoxylin stain channel of image tile #28943 at 40X magnification from subject TCGA-AR-A5QQ with genotype: GG_AA for rs17320521 by rs869096886. (**b**) Hematoxylin stain channel of image tile #6545 at 40X magnification from subject TCGA-EW-A1PB with genotype: AA_GG for rs17320521 by rs869096886. (**c**) Histograms of log2 size area of the nuclei in the images from subject TCGA-AR-A5QQ with genotype: GG_AA for rs17320521 by rs869096886 and subject TCGA-EW-A1PB with genotype: AA_GG for rs17320521 by rs869096886. The x-axis is log2 size area and the y-axis is the density of the data. (**d**) Same as Fig. 1e except for TCGA *WSCD2* gene log2 (RSEM + 1) on the y-axis and 284 of the 286 patients. p-values for the comparisons are from Mann–Whitney tests with the hypothesis that the median *WSCD2* gene expression of the patients with genotype homozygous for the minor alleles is the same as the other genotypes and the alternative is that the median is less. The dotted line is the median expression of *WSCD2* in TCGA normal patients. (**e**) Table with median values of *WSCD2* gene log2 (RSEM + 1) for sample groups. (**f**) t-test comparisons of RNA-Seq FPKM gene expression data between TCGA patients with genotype homozygous for the minor alleles for of rs17320521 by rs869096886 versus all other genotypes. For each comparison, the number of differentially expressed genes (DEGs) is shown based on a false discovery rate < 0.05 and fold change >|1.5|. (**g**) Interaction networks derived from the 16 genes from the overlap of the DEGs in (**f**), *WSCD2* and *MT-ND4* using the Ingenuity Pathway Analysis Knowledge Base content version 62,089,861. These focus genes are shaded gray. Square: cytokine, diamond: enzyme, triangle: kinase, horizontal oval: transcription regulator, horizontal rectangle: ligand-dependent nuclear receptor, double circle: complex, single circle: other. (**h**) Same as (**g**). except the interaction networks derived from the 9 of the 16 genes from the overlap of the DEGs in (**f**), *WSCD2* and *MT-ND4*. Vertical rectangle: G-protein coupled receptor, vertical oval: transmembrane receptor, hexagon: translation regulator.

Comparison of two patches from images of H&E slides from a TCGA breast cancer patient (TCGA-AR-A5QQ) homozygous for major alleles of the SNPs (Fig. 2a) and a TCGA breast cancer patient (TCGA-EW-A1PB) homozygous for the minor alleles of the SNPs (Fig. 2b) visually depicts the morphological change in the size increase of the nuclei associated with the variants. As shown by the histogram of the Size Area of the nuclei (Fig. 2c) and analysis of the data (Supplementary Data 6), the mean Size Area per nuclei = 4.7 (SD = 1.02) for the image from TCGA-EW-A1PB which is about 23% larger than that of TCGA-AR-A5QQ (mean Size Area per nuclei = 3.6, SD = 1.26). Patch-wise, the TCGA-AR-A5QQ image tile #: 28943 Size Area per nuclei is 13.03 whereas the TCGA-EW-A1PB image tile #: 6545 Size Area per nuclei is 26.90 (~ 52% larger).

### Gene expression analysis reveals gene interactions

Analysis of RNA-Seq data from the TCGA Splicing Variants Database ^6^ shows a decrease in RNA-Seq by Expectation Maximization (RSEM) expression of *WSCD2* in the TCGA breast cancer patients that are homozygous for the minor alleles of the SNPs (Fig. 2d). However, only one comparison (AA_GG vs GA_GG) is significant (p-value < 0.05). The median expression for each sample group is shown in Fig. 2e. The median decrease of *WSCD2* gene expression in the TCGA breast cancer patients that are homozygous for the minor alleles of the SNPs is ~ 1.7 fold versus the TCGA breast cancer subjects that are heterozygous for the nuSNP alleles and homozygous for the minor allele of the mtSNP (Fig. 2d).

**Figure 3:**
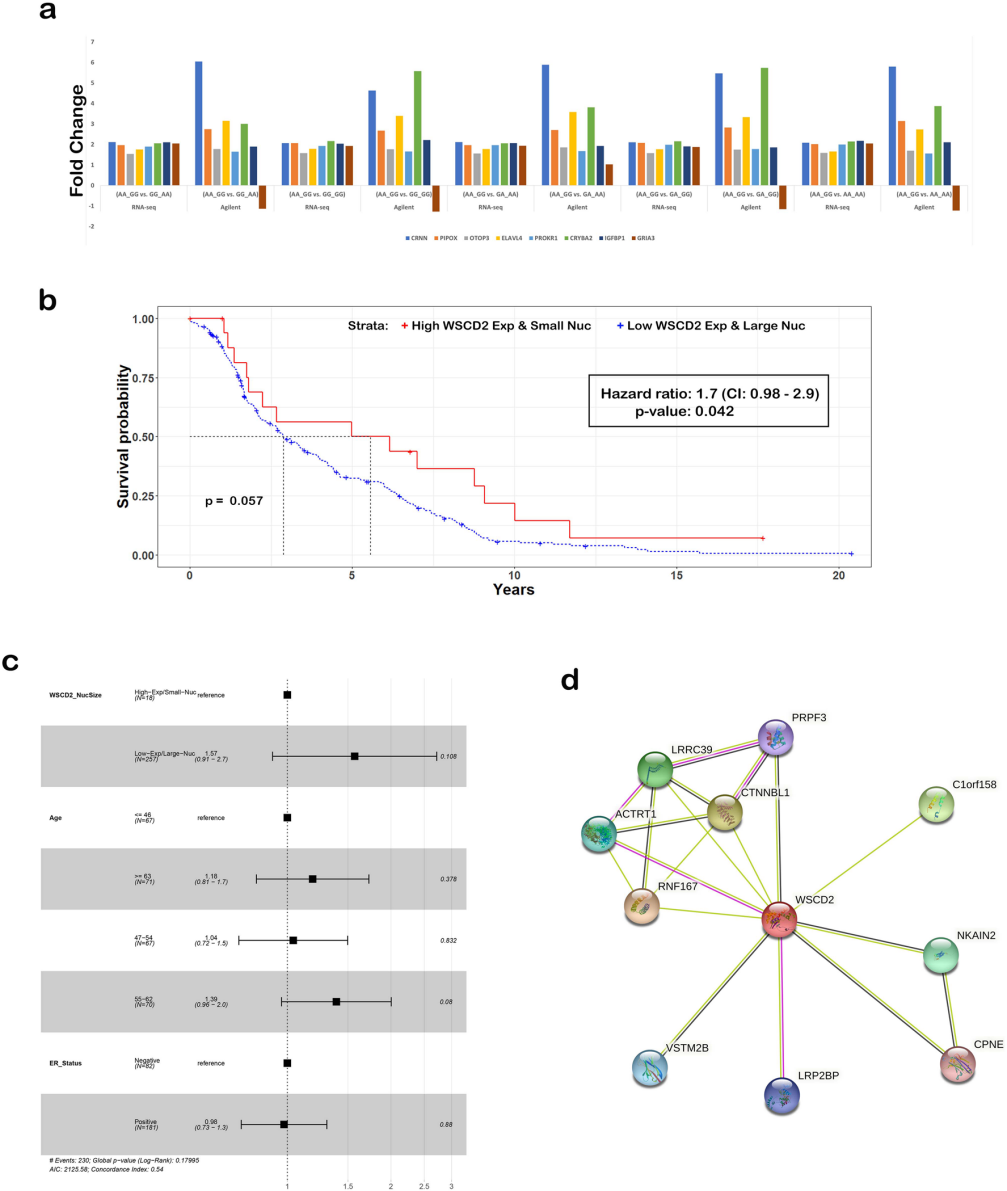
Gene expression validation, survival analysis and proteins interacting with *WSCD2*. (**a**) Validation of 8 of the 16 RNA-Seq DEGs using Agilent gene expression data (log2 lowess normalized (cy5/cy3) collapsed by gene symbol) from 130 of the 286 TCGA patients that were available in the U.S. National Institutes of Health National Cancer Institute Genomic Data Commons legacy archive. The y-axis is fold change and the x-axis is the gene colored according to the legend as well as grouped by the comparison using either data from RNA-Seq or Agilent microarray. (**b**) Kaplan–Meier plot of time-to-event survival from 277 of the 286 TCGA breast cancer female patients on their bulk RNA-Seq RSEM expression of *WSCD2* and nucleus mean Size Area data. The x-axis is years and the y-axis is survival probability. The red curve is the data for patients with high *WSCD2* gene expression (> the 75th percentile) and small nuclei (< the 25th percentile) and the blue curve is the data from the other patients (low *WSCD2* gene expression and large nuclei). The dashed black lines are the medians of survival for each strata and p is the log-rank p-value from testing the null hypothesis that each strata has the same survival probability. The hazard ratio value and statistical inferences for the parameter is shown in the inset. (**c**) Forest plot for Cox proportional hazards model. N is the number of patients, the hazard ratio is represented by the square, its confidence interval in parentheses and is represented by the horizontal lines, the vertical line represents 1.0 and the p-value for each predictor variable is to the right of the plot. (**d**) Search Tool for the Retrieval of Interacting Genes/Proteins (STRING) database^16^ network of proteins that interact with the WSCD2 protein. Nodes are filled to denote that the 3D structure of the protein is known or predicted. Pink interactions are experimentally derived whereas lime or black colored interactions are derived by text mining literature or by gene co-expression analysis respectively.

To identify other genes that are differentially expressed in the TCGA patients homozygous for the minor alleles of the *WSCD2* gene nuSNP rs17320521 interacting with *MT-ND4* gene mtSNP rs869096886, we performed analysis of variance modeling with post-hoc pairwise t-tests of the genes to compare with those in the TCGA patients with the other genotypes (Fig. 2f). Differentially expressed genes (DEGs) were identified based on a false discovery rate < 0.05 and fold change >|1.5| with 16 genes in common between the comparisons (Supplementary Data 7). Network analysis of the DEGs revealed several core interactions (direct or indirect) with *MT-ND4* that are related to growth factors and transcription factors (Fig. 2g), as well as those specifically associated with insulin (Fig. 2h). These findings are interesting, as recently insulin resistance has been associated with breast cancer incidence and mortality^7^. Of the 286 TCGA patients, 130 had Agilent microarray gene expression data in the U.S. National Institutes of Health National Cancer Institute Genomic Data Commons legacy archive. Based on 8 of the 16 RNA-Seq DEGs that were on the array (Supplementary Data 8), we validated that 7 of the genes have comparable expression to the RNA-Seq data in terms of fold change direction (Fig. 3a and Supplementary Data 9). In all but one of the comparisons, the glutamate ionotropic receptor AMPA type subunit 3 (*GRIA3*) gene fold change is in the opposite direction of the RNA-Seq data. In addition, the Agilent gene expression fold change values for cornulin (*CRNN*), pipecolic acid and sarcosine oxidase (*PIPOX*), ELAV like RNA binding protein 4 (*ELAVL4*), and crystallin beta A2 (*CRYBA2*) are higher than that of the RNA-Seq.

### Low expression of *WSCD2* coupled with large nuclei is associated with an increased risk of mortality

To assess the impact of *WSCD2* gene expression and the size of the nuclei in terms of survival, we perform a Cox regression analysis of the right censored, time-to-event survival outcome from 277 of the 286 TCGA female breast cancer patients (Supplementary Data 10). The data for each patient was grouped according to high *WSCD2* gene expression and small nuclei, if the gene expression is > the 75th percentile and the mean Size Area is < the 25th percentile, otherwise grouped as low *WSCD2* gene expression and large nuclei. The Cox proportional hazards ratio = 1.7 (confidence interval: 0.98–2.9) with a p-value = 0.042 suggests that having low *WSCD2* gene expression and large nuclei can be interpreted as a 70% increase in risk of death. The Kaplan–Meier plot of the data reveals a borderline significant (p-value = 0.057) increased risk of mortality for the TCGA patients with low *WSCD2* gene expression and large nuclei versus those with high *WSCD2* gene expression and small nuclei (Fig. 3b). When age of the patients at diagnosis (dichotomized into four groups) and estrogen receptor (ER) status were added as covariate to the Cox regression model, the hazard ratio = 1.57 (confidence interval: 0.91–2.7) and was not significant (p-value = 0.108) for low *WSCD2* gene expression and large nuclei (Fig. 3c).

## Discussion

Mitochondria evolved over time to communicate with the nuclear genome in order to perform critical biological functions such as cellular energy production, oxidation–reduction processes and modulation of apoptosis^8^. The human and rodent mitochondrial genomes are circular, double-stranded DNA, of approximately 16.5 kb in length and contains 37 genes coding for rRNAs, tRNAs and polypeptides. The nuclear genome encodes for several mitochondrial proteins^9^ and conversely, the mitochondria has been shown to impact epigenetic marks or mutations in the nuclear genome^10^. In fact, mito-nuclear interactions have been shown to contribute to the regulation of genes as part of the cell’s inter-organelle communication^11^ and that these interactions are not random occurrences nor merely incidental^12^. Hence, maintenance of unaltered mito-nuclear epistasis is paramount to optimal fitness in species. Nevertheless, mutations in one or both of the genomes lead to biological conditions, phenotypic changes and disease states^13^.

In this study we sort out to investigate the epistatic relationship between nuSNPs in or flanking genes that encode mitochondrial “bioenergetic” proteins and mtSNPs in breast cancer. Using a mixed effect linear model with a kinship matrix as the covariance structure to capture the relatedness of the subjects, we identified a nuSNP and a mtSNP that jointly increased the size of nuclei in H&E images of breast cancer tissue from TCGA and METABRIC cohorts as much as 12% (Fig. 1e,f). Physicians and pathologist include the shape, size and amount of DNA in nuclei as part of grading the severity of breast cancer. Previously, nuclear morphological features have been correlated with ductal carcinoma in situ of the breast in Singapore women ^14^. Our results show that variants in genes in the mitochondrial genome may be playing a key role jointly with variants in the nuclear genome that could be further explored for assessing susceptibility to breast cancer.

The only significant mito-nuclear interaction that was confirmed to increase the size of nuclei is between nuSNP rs17320521 in an intron of the WSC Domain Containing 2 (*WSCD2*) gene and mtSNP rs869096886, a synonymous variant mapped to the mitochondrially-encoded NADH dehydrogenase 4 (*MT-ND4*) gene. Analysis of bulk RNA-Seq revealed a decrease in *WSCD2* gene expression in the samples from TCGA breast cancer patients with the genotype homozygous for the minor alleles (Fig. 2d,e) and core gene networks of transcription factors, growth factors and insulin indirectly associated with *MT-ND4* (Fig. 2g,h). Incidentally, a variant in *WSCD2* has been found to be associated with insulin sensitivity^15^. These findings are intriguing as recently insulin resistance has been associated with breast cancer incidence and mortality^7^.

Interestingly, the Search Tool for the Retrieval of Interacting Genes/Proteins (STRING) database^16^ contains several interactions with WSCD2 that are proteins functioning as either tumor suppressors, promotors of tumorigenesis or prognosticators of various forms of cancers (Fig. 3d). In fact, the expression of LDL receptor-related proteins (LRPs) in solid malignancies has recently been shown to correlate with cancer survival^17^. These associations, coupled with the increase in risk of death depending on low *WSCD2* expression and large nuclei (Fig. 3b), warrants further research investigation of the gene and the potential role it may play in breast cancer.

To date, WSCD2 has no known function and there is not sufficient biological information to theorize about a possible mechanism of how the downregulation of *WSCD2* gene expression may be playing a role in affecting nuclei size or in what way the mito-nuclear interaction is contributing to breast cancer pathogenesis*.* However, there are a few hypotheses about the mechanisms involved in regulating the size of nuclei in cancer. Jevtić and Levy (2014) provide a detailed review of molecular mechanisms controlling nuclear size in model system organisms and they speculate how nuclear size might contribute to cancer development and progression^18^. Recently, Mukherjee et al. (2020) used sea urchin embryos to discover that the perinuclear endoplasmic reticulum plays a role in scaling nuclear size independent of the size of the cell during early development^19^. Additional research from the Levy lab suggested that the nuclear-to-cytoplasmic volume ratio was shown to increase in HeLa and MRC-5 cancer cell lines by manipulation of the levels of importin α2 (IMPα2) and nuclear transport factor 2 (NTF2)^20^. As more investigation is dedicated to underlying the mechanisms of the mito-nuclear interaction of the variants in *WSCD2* and *NT-ND4* and its association with increased nuclei size, the closer we will be to a better understanding of the complexity of breast cancer and devising novel therapeutics to treat and/or cure the disease.

## Methods

All experiments were performed in accordance with relevant named guidelines and regulations. Informed consent was obtained from all participants and/or their legal guardians to analyze the contributed data and images as well as to publish the results of analyses and images in an online open-access publication. The databases used to access data are all publicly available but require authorization to obtain controlled data. TCGA data were in whole or part based upon studies approved and generated by the TCGA Research Network: https://www.cancer.gov/tcga. The GTEx Project data was based upon studies approved, supported and generated by the Common Fund of the Office of the Director of the National Institutes of Health (commonfund.nih.gov/GTEx). The METABRIC project data was based on studies approved, supported and funded in whole or part by Cancer Research UK, the British Columbia Cancer Foundation and Canadian Breast Cancer Foundation BC/Yukon. These data are publicly available upon authorized access and are approved for general research use by respective data access committees (TCGA: U.S. National Cancer Institute; GTEx: U.S. National Human Genome Research Institute; METABRIC: Cancer Research UK Cambridge Institute). Thus, an Institutional Review Board (IRB) was not required for data access and analysis.

### Breast tissue images

Two hundred and ninety TCGA breast cancer H&E stained pathology whole slide image (WSI) .svs files of formalin-fixed paraffin-embedded sections of breast mammary tissue were downloaded from The Cancer Imaging Achieve (TCIA) associated with the data base of Genotypes and Phenotypes (dbGaP) accession phs000178.v11.p8. In addition, a set of 886 non-diseased GTEx project breast mammary tissue H&E pathology WSI .svs files were downloaded from the National Human Genome Research Institute (NHGRI) Analysis, Visualization and Informatics Lab-space (AnVIL) TERRA cloud workspace associated with dbGaP accession phs000424.v8.p2. As an independent data set, H&E stained pathology breast cancer .jpg image patches from WSIs of 564 breast cancer subjects as part of METABRIC were downloaded from the European Genome-Phenome Archive (EGA) under dataset identifier EGAD00010000270. The WSIs are Aperio format, with the TCGA breast cancer and GTEx images containing single-file pyramidal TIFFs of 1024 × 768 pixels with tiles of size 240 × 240 pixels while the METABRIC patches were filtered for images of size at least 1986 × 1986 pixels.

### Image processing to extract morphological phenotypes

The HistomicsTK python package v0.10 was used to preprocess the images and extract morphological features of the tissues as phenotypic measurements. Briefly, each tile within an image was checked to determine if it was overly black (85th percentile of the pixels < 15) or white (15th percentile of the pixels > 240) in order to exclude it. Then, each tile H&E stain color was deconvoluted to extract the hematoxylin stained channel and with a threshold of ½ the mean pixel values, the foreground (pixels with values ≥ threshold = 1) was delineated from background (pixels with values < threshold = 0). Using a minimum radius of 10, a maximum radius of 15 and a local max search radius of 10, the foreground was masked to filter out small objects and then segmented to detect nuclei that are resolved for overlapping regions of interest and holes. For each tile, the following morphology features and estimates of the nuclei were extracted for computation of morphological phenotypes:

Number of nuclei = # of segment objects in the foreground.

Size Area = # of pixels of the objects in the foreground.

Intensity = mean pixel intensities of the objects in the foreground.

Circularity = (4*pi*area)/perimeter^2^, where perimeter is the contour line of each object in the foreground through the centers of border pixels using a 4-connectivity. That is, in terms of pixel coordinates, every pixel that has the coordinates (x ± 1, y) or (x,y ± 1) is connected to the pixel at (x,y).

The mean of the Size Area and Intensity were divided by the mean number of nuclei and then log2 transformed.

### Genotype data acquisition

TCGA breast cancer: Legacy genotype data for 2,263 TGCA breast cancer patients in birdseed format was downloaded from the Genomic Data Commons (GDC) associated with the dbGaP accession phs000178.v10.p8. The .txt files were converted to variant call format (vcf) files of SNPs from the Affymetrix GenomeWideSNP_6 human SNP chip array release n35 with the hg19 (GRCh37-lite) genome reference and dbSNP v141. There were 286 TGCA breast cancer female patients that had corresponding morphological features extracted from the image data.

GTEx: Genotype vcf files of SNPs (dbSNP v150) called via Illumina whole genome sequencing (hg38) of the nuclear genome and mitochondrial genome from 866 and 979 GTEx subjects respectively were downloaded from the AnVIL TERRA cloud workspace (dbGaP Study Accession: phs000424.v8.p2). There were 259 GTEx female donors that had corresponding morphological features extracted from the image data.

METABRIC: Affymetrix GenomeWideSNP_6 human SNP chip array release n35 (hg19 and dbSNP v141) .CEL files for 543 METABRIC BRCA subjects were downloaded from the EGA under dataset identifier EGAD00010000266. The genotypes were called using the apt-probeset-genotype executable in Thermo Fisher Scientific Analysis Power Tools release 2.10.2.2 and the Affymetrix GenomeWideSNP_6 chip description file, the precomputed birdseed-v2 SNP specific reads model as well as the following arguments: –set-gender-method: cn-probe-chrXY-ratio, –chrX-probes: GenomeWideSNP_6.chrXprobes, –chrY-probes: GenomeWideSNP_6.chrYprobes, –special-snps: GenomeWideSNP_6.specialSNPs. There were 523 METABRIC female subjects that had corresponding morphological features extracted from the image data.

### Genotype data filtering, analysis, annotation and visualization

Autosomal nuSNPs with a call rate < 95%, Hardy–Weinberg Equilibrium (HWE) < 0.001 or minor allele frequency (MAF) < 0.05 (according to the 1000 genomes phase 3 genotype as a reference) were removed. SNPs on the sex chromosomes were not considered for analysis. Any nuSNP with missing genotype data for a subject was imputed according to the frequency distribution of the alleles for that SNP from the other subjects. nuSNPs were coded according to the number of minor alleles (0,1,2). We focused on the ~ 12,400 nuSNPs that mapped within or flank genes that encode mitochondrial proteins as determined by the MitoMiner v4.0^21^ and MitoCarta v2.0^22,23^ databases. mtSNPs with variant allele frequency (VAF) < 0.05 were removed. mtSNPS were coded as to whether or not the minor allele was present (0 is not having the minor allele or 1 having the minor allele). SNPs were annotated with SnpEff^24^ version 4.4 using the human hg38 genome version. A kinship matrix for each cohort containing the resemblance of the female subjects was generated from their nuSNP genotype data using the efficient mixed model association (EMMA) R package v1.1.2^25^ with additive genetic effect.

### Mito-nuclear interaction test

The following mixed linear model constructed with the lmekin function in the COXME R package v2.2–10 was used to test the joint effect of nuSNPs and mtSNPs on each phenotype

$${Y}_{ijkl}={\beta }_{0}+{\beta }_{1}{N}_{i}+{\beta }_{2}{M}_{j}+{\beta }_{3}(NM{)}_{ij}+{S}_{{0}_{k}}+\sum_{c=1}^{5}{\beta }_{{P}_{c}}{P}_{c}+{\varepsilon }_{ijkl}$$

where *Y*_*ijkl*_ is the log base 2 of the *l*th phenotype observation, $\beta$_0_ is the grand mean, *N*_*i*_ is the *i*th genotype of the nuclear SNP, *M*_*j*_ is the *j*th genotype of the mitochondrial SNP, *S*_0,*k*_ is a random effect for the *k*th subject fitted with a kinship matrix as the covariance structure to account for the relatedness of the subjects, *P*_*c*_ is the *c*th principal component as a covariate to adjust the *l*th phenotype for population structure and *e*_*ijkl*_ is the error term. The null hypothesis H_0_ is $\beta$_3_ = 0 and the alternative H_a_ is $\beta$_3_ ≠ 0. Let the chi-square likelihood ratio test statistic D =  − 2(ln(likelihood null model)–ln(likelihood full model)) where the null model is the mixed model without the interaction term and the full model is the one with the interaction term. The p-value for each association of a mtSNP-nuSNP joint effect on a phenotype was obtained from the distribution of D≈χ^2^ with 1 degree of freedom. Multiple testing was controlled by false discovery rate (FDR) adjusted p-values (q-values^26^) where the proportion of true null hypotheses (pi0) was estimated by bootstrapping the p-values. Significant interactions (q-value < 0.05) were further evaluated with one-sided Mann–Whitney *U* post hoc tests of the population (U) of the phenotype for each genotype pair (H_o_: *U*_1_ = *U*_2_), H_a_: *U*_1_ > *U*_2_). The distribution of the phenotype of the samples with the minor alleles from the nuSNP and mt SNP (*U*_1_) was required to have 1) a Bonferroni p-value < 0.05 for each comparison vs the distribution of the phenotype of the samples with the other genotypes (*U*_2_) and 2) a distribution stochastically greater than *U*_2_.

### Cox proportional hazards analysis

The survival package in R^27,28^ was used to perform a Cox regression analysis of the right censored, time-to-event survival outcome from 277 of the 286 TGCA breast cancer female patients on their bulk RNA-Seq RSEM expression of *WSCD2* and nucleus mean Size Area data. The data for each patient was coded into a categorical predictor variable with two levels: high-expression and small-nuclei, if the gene expression is > the 75th percentile and the mean Size Area is < the 25th percentile, otherwise coded as low-expression and large-nuclei. The Cox proportional hazards likelihood ratio test statistic and a Chi-square distribution with 1 degree of freedom were used to assess the statistical significance of the model. In addition, a multivariate survival analysis was performed using the Cox regression model with the ER status of 275 patients (indeterminates not included) and their age at diagnosis (dichotomized into four groups according to quantiles) included as covariates, and significance based on a Chi-square distribution with 5 degrees of freedom.

## Supplementary Information

Supplementary Figure 1.

Supplementary Figure 2.

Supplementary Information.

Supplementary Data 1.

Supplementary Data 2.

Supplementary Data 3.

Supplementary Data 4.

Supplementary Data 5.

Supplementary Data 6.

Supplementary Data 7.

Supplementary Data 8.

Supplementary Data 9.

Supplementary Data 10.

## Data Availability

TCGA breast cancer genotype and gene expression data are available from the GDC associated with the dbGaP accession phs000178.v10.p8. TCGA breast cancer images are available from TCIA associated with the dbGaP accession phs000178.v11.p8. GTEx genotype and images are available from the AnVIL TERRA cloud workspace associated with dbGaP Study accession phs000424.v8.p2. The METABRIC genotype data files and images were downloaded from the EGA under dataset identifiers EGAD00010000266 and EGAD00010000270 respectively.

## Code availability

Python modules to extract morphological features from the H&E images are available in the HistomicsTK package. Software to call genotype data from Affymetrix GenomeWideSNP_6 human SNP chip array CEL files is available in the Thermo Fisher Scientific Analysis Power Tools. The code for mixed linear modeling of the mito-nuclear interaction data is available through the lmekin function in the COXME R package.
