# Supplementary figures and images for: Mitochondrial-nuclear epistasis underlying phenotypic variation in breast cancer pathology
Source: Sci Rep. 2022 Jan 26;12:1393. doi: 10.1038/s41598-022-05148-4 (PMC8791930; doi:10.1038/s41598-022-05148-4)

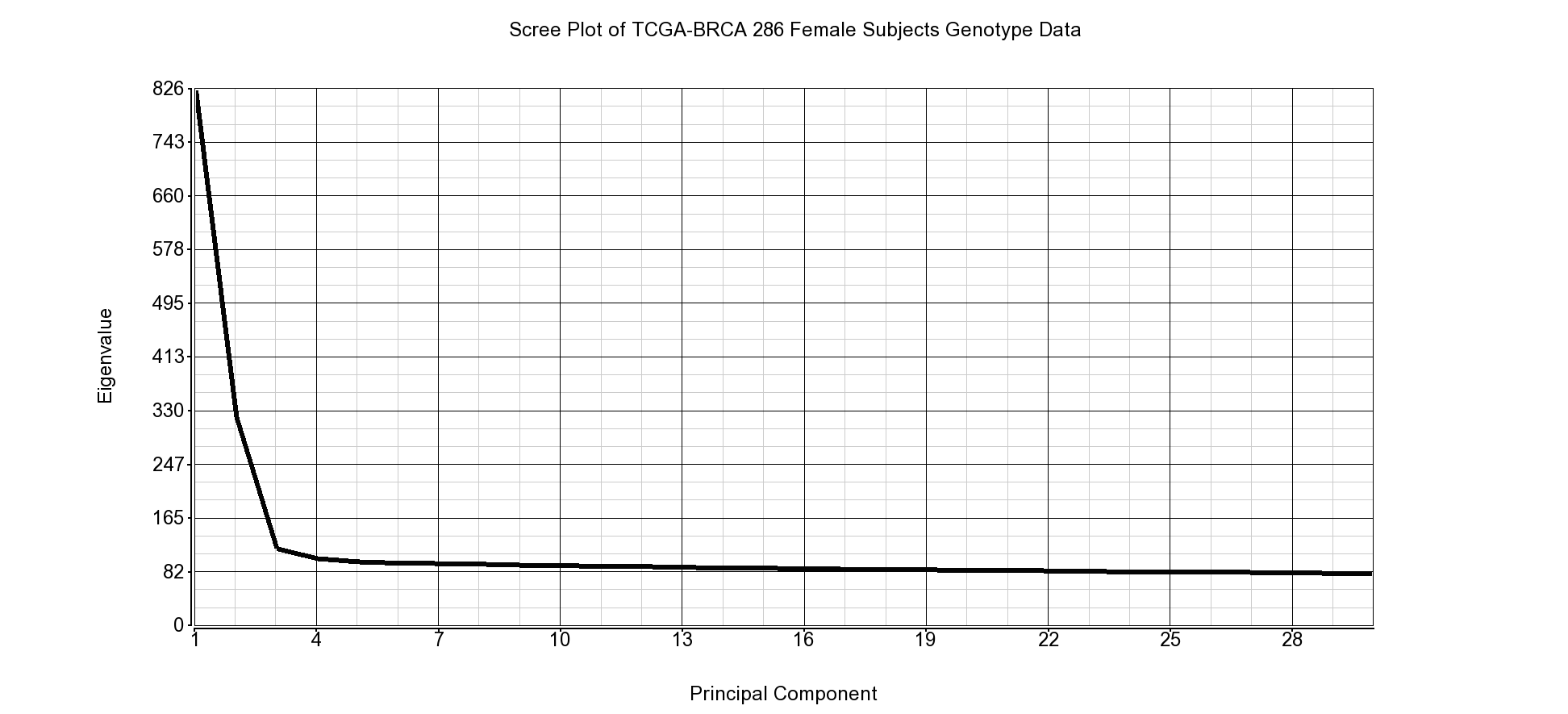

Supplement: Supplementary file 1 — Supplementary Figure 1. [file 41598_2022_5148_MOESM1_ESM.tiff]

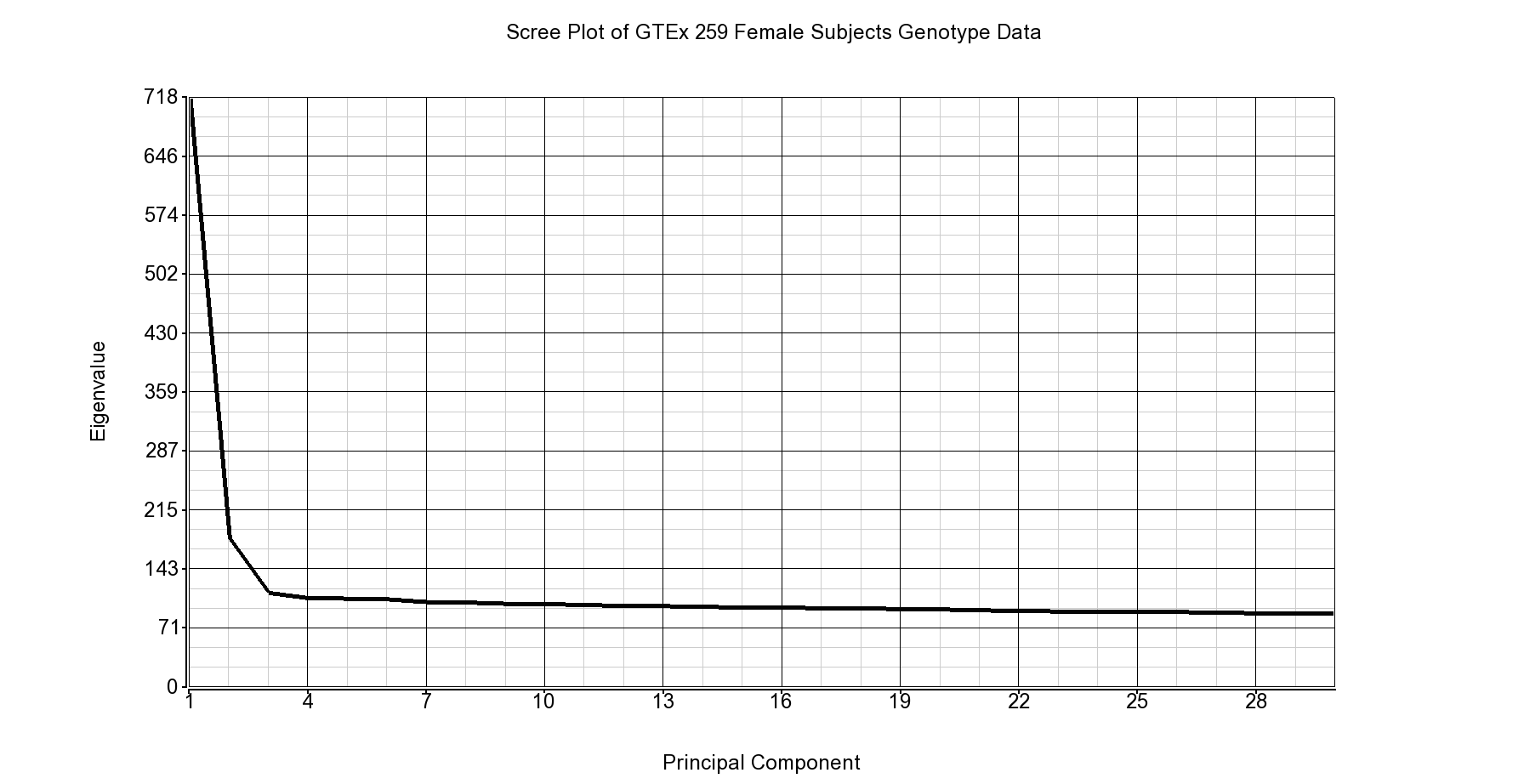

Supplement: Supplementary file 2 — Supplementary Figure 2. [file 41598_2022_5148_MOESM2_ESM.tiff]
