# Supplementary material for: Mitochondrial-nuclear epistasis underlying phenotypic variation in breast cancer pathology
Source: Sci Rep. 2022 Jan 26;12:1393. doi: 10.1038/s41598-022-05148-4 (PMC8791930; doi:10.1038/s41598-022-05148-4)
Supplement: Supplementary file 3 — Supplementary Information. [file 41598_2022_5148_MOESM3_ESM.docx]

Legend to Supplementary Figure 1. Scree plot of TCGA genotype data. Principal component analysis performed on the genotype data from 286 TCGA breast cancer female patients was performed. The x-axis is the 1st 30 principal components and the y-axis is the eigenvalues.

Legend to Supplementary Figure 2. Scree plot of GTEx genotype data. Principal component analysis performed on the genotype data from 259 GTEx non-diseased female subjects was performed. The x-axis is the 1st 30 principal components and the y-axis is the eigenvalues.
